# Single‐cell transcriptomics reveal metastatic *CLDN4*+ cancer cells underlying the recurrence of malignant pleural effusion in patients with advanced non‐small‐cell lung cancer

**DOI:** 10.1002/ctm2.1649

**Published:** 2024-04-17

**Authors:** Xiaoshen Zhang, Xuanhe Wang, Yaokai Wen, Shen Chen, Caicun Zhou, Fengying Wu

**Affiliations:** ^1^ School of Medicine Tongji University Shanghai China; ^2^ Department of Medical Oncology Shanghai Pulmonary Hospital, Tongji University School of Medicine Shanghai China

**Keywords:** disseminated tumour cells, malignant pleural effusion, metastasis, non‐small‐cell lung cancer, predictive marker, single‐cell RNA sequencing

## Abstract

**Background:**

Recurrent malignant pleural effusion (MPE) resulting from non‐small‐cell lung cancer (NSCLC) is easily refractory to conventional therapeutics and lacks predictive markers. The cellular or genetic signatures of recurrent MPE still remain largely uncertain.

**Methods:**

16 NSCLC patients with pleural effusions were recruited, followed by corresponding treatments based on primary tumours. Non‐recurrent or recurrent MPE was determined after 3–6 weeks of treatments. The status of MPE was verified by computer tomography (CT) and cytopathology, and the baseline pleural fluids were collected for single‐cell RNA sequencing (scRNA‐seq). Samples were then integrated and profiled. Cellular communications and trajectories were inferred by bioinformatic algorithms. Comparative analysis was conducted and the results were further validated by quantitative polymerase chain reaction (qPCR) in a larger MPE cohort from the authors' centre (*n* = 64).

**Results:**

The scRNA‐seq revealed that 33 590 cells were annotated as 7 major cell types and further characterized into 14 cell clusters precisely. The cell cluster C1, classified as Epithelial Cell Adhesion Molecule (*EpCAM*)+ metastatic cancer cell and correlated with activation of tight junction and adherence junction, was significantly enriched in the recurrent MPE group, in which Claudin‐4 (*CLDN4*) was identified. The subset cell cluster C3 of C1, which was enriched in recurrent MPE and demonstrated a phenotype of ameboidal‐type cell migration, also showed a markedly higher expression of *CLDN4*. Meanwhile, the expression of *CLDN4* was positively correlated with E74 Like ETS Transcription Factor 3 (*ELF3*), *EpCAM* and Tumour Associated Calcium Signal Transducer 2 (*TACSTD2*), independent of driver‐gene status. *CLDN4* was also found to be associated with the expression of Hypoxia Inducible Factor 1 Subunit Alpha (*HIF1A*) and Vascular Endothelial Growth Factor A (*VEGFA*), and the cell cluster C1 was the major mediator in cellular communication of *VEGFA* signalling. In the extensive MPE cohort, a notably increased expression of *CLDN4* in cells from pleural effusion among patients diagnosed with recurrent MPE was observed, compared with the non‐recurrent group, which was also associated with a trend towards worse overall survival (OS).

**Conclusions:**

*CLDN4* could be considered as a predictive marker of recurrent MPE among patients with advanced NSCLC. Further validation for its clinical value in cohorts with larger sample size and in‐depth mechanism studies on its biological function are warranted.

**Trial registration:**

Not applicable.

## Graphical abstract

**Highlights**

- High level of *CLDN4*+ cancer cells can mediate the recurrence of malignant pleural effusion in advanced NSCLC patients.
- *CLDN4* may contribute to cancer cell proliferation and is regulated by transcription factor *ELF3*.
- *MIF* signaling and *VEGFA* signaling shaped the immunosuppressive microenvironment in pleural effusion, contributing to the recurrence of MPE among advanced NSCLC patients.

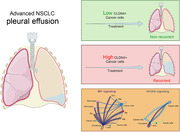

## 1 BACKGROUND

Malignant pleural effusion (MPE) refers to the accumulation of pleural fluid caused by thoracic malignancies and still lacks standard managements currently.^2^ Patients have a median survival of only 4−7 months from the time of diagnosis and usually suffer from severe respiratory distress due to the presence and recurrence of MPE.^3^, ^4^, ^5^ Removing fluid and preventing its accumulation is presently the main strategy for the management of MPE.^6^, ^7^ Among patients with non‐small‐cell lung cancer (NSCLC), the initiation of MPE usually marks the advanced disease stage, occurring in more than 15% of patients during the course of the disease. Thus, palliative treatments, including thoracentesis, pleurodesis, and indwelling pleural catheterization,^6^ are more practical and favourable for MPE treatments in clinical practice,^8^ with the aim of improving patients’ quality of life while controlling the frequency of invasive procedures.^8^ However, studies utilizing these palliative therapies still did not yield satisfactory outcomes for patients with MPE,^9^, ^10^, ^11^ especially for those with recurrent one.

Conventionally, lymphatic obstruction, increased vascular permeability, and inflammations were considered the main mechanisms of MPE formation.^12^, ^13^ Nevertheless, recent discoveries have challenged such limited understandings, and continuously expand them. For example, Giannou et al. suggested that mast cells could be recruited by tumour cells to assist in forming pleural effusion.^14^ Meanwhile, Niu et al. implied Th17 as a vital player in the development of MPE, and Wu et al. further found a positive relationship between naïve B cells and Th17.^15^, ^16^ In addition, the importance of Vascular Endothelial Growth Factor (*VEGF*) signalling in MPE generation was also pinpointed.^17^ Through analysing the clinical outcomes of patients with treatments, Hsu et al. suggested that Tumour Necrosis Factor Alpha (*TNF‐α*) elevation was observed in patients with unsuccessful pleurodesis.^18^ Wang et al. also indicated that macrophage‐derived C‐C Motif Chemokine Ligand 22 (*CCL22*) influenced the secretion level of Transforming Growth Factor Beta (*TGF‐β*) and suppressed the immune activity.^19^ Practically, many studies yielded significant findings in mice models,^20^, ^21^ but there are fundamental differences between mice and human, such as the thoracic structures and the amount of produced pleural fluid. Besides, the MPE mice model was established by direct pleural injection of cancer cells as a simulation of intrapleural metastasis, while in real clinical settings, cancer cells went through a series of complex mechanisms, including invasion and migration from tumour primary lesion to pleura.^22^ Consequently, the value of findings from the mice models needs to be further evaluated, and we still lack effective strategies for the diagnosis of MPE and the prediction for its recurrence in clinical practice.

More than 50% of MPE will recur within 90 days.^23^ Mechanically, recurrent MPE represents a deteriorating state of advanced NSCLC. MPE stands for a complex ecosystem with tumour cells and immune cells, in which their interaction plays the main role. In recurrent MPE, the interaction between these cells becomes even more heterogeneous and leads to a cascade effect, which eventually results in the recurrence of MPE. Several mechanisms have been reported to further lead to the progression or recurrence of MPE. For instances, Luo et al. revealed that the complement system could recruit the monocytes and lead to MPE progression.^24^ Hardak et al. suggested that human heparinase could facilitate the anticoagulant microenvironment in MPE and cause the pleural fluid recurrence.^25^ Nosti et al. also clarified how Colony Stimulating Factor 1 (*CSF1*) augmented vascular permeability and destabilized tumour vessels to regenerate MPE.^26^ In addition, Wang et al. suggested a Forkhead Box P3 (*FOXP3*)+ natural killer T‐like cell could suppress the immune microenvironment in recurrent MPE.^27^ Furthermore, during the pathogenic process of recurrent MPE, it is well‐acknowledged that cancer cells are the initiating factor. Cancer cells in pleural effusion highly express adhesive molecules, which are closely related to their metastasis from the primary lesions. This procedure has not been clearly elucidated so far. Therefore, defining the molecular phenotypes of metastatic tumour cells in recurrent pleural effusion is helpful to further predict and diagnose recurrent MPE among NSCLC patients.

In our study, we found that the cancer cells in the pleural effusion exhibited the phenotype of circulating tumour cells (CTCs) and expressed metastatic markers such as Epithelial Cell Adhesion Molecule (*EpCAM*) and Tumour Associated Calcium Signal Transducer 2 (*TACSTD2*). Moreover, we introduced Claudin‐4 (*CLDN4*), a gene that expressed on cancer cells, had the potential to be the predictive biomarker of recurrent MPE among advanced NSCLC patients. *CLDN4* belongs to claudins, the most significant constituents of tight junctions.^28^ The overexpression of *CLDN4* has been reported in various cancers, including gastric cancer, pancreatic cancer, colorectal cancer, breast cancer, oral squamous cell carcinoma, ovarian cancer, bladder cancer, cholangiocarcinoma, and NSCLC. In all of these cases, *CLDN4* expression correlated with disease progression and poor prognosis, which was speculated to be related to its function in carcinogenesis, barrier function and maintenance of intratumoural microenvironment, apoptosis, stemness and epithelial‐mesenchymal transition (EMT). However, it was less discussed in the association with lung cancer under the condition of recurrent MPE. In this study, we performed single‐cell RNA sequencing (scRNA‐seq) on MPE samples and revealed the positive correlation between *CLDN4* and recurrent MPE. The expression pattern of *CLDN4* on circulating cancer cells denoted its potential for early diagnosis or prediction of recurrent MPE, which also shed lights on the therapeutic strategy of recurrent MPE for advanced NSCLC patients.

## 2 METHODS

### 2.1 Patient cohort and pleural effusion sample collection

We prospectively enrolled 16 NSCLC patients with pleural effusion into this study, and confirmed that all these patients had MPE through computer tomography (CT) and pathology. Baseline pleural fluid samples were then collected for subsequent sequencing and analysis. For lung adenocarcinoma patients with Epidermal Growth Factor Receptor (*EGFR*) or Anaplastic Lymphoma Kinase (*ALK*) mutations, appropriate targeted therapy strategies were used. Patients with lung squamous carcinoma were treated with platinum‐based chemotherapy strategies. Previously researchers classified patients with recurrent MPE into groups based on their choice of second pleural procedure within two weeks of the first thoracentesis and found that MPE would recur within the first two weeks among roughly one third of all patients.^29^ Meanwhile, another study claimed that at day 15, 30, 60 and 90, the pleural fluid recurrence rate of patients with malignancy was 30%, 40%, 45% and 48%, respectively.^30^ Due to the high early recurrence rate, taken together, we considered 3−4 weeks could be a clinically feasible cut‐off time to distinguish the recurrent or non‐recurrent MPE. In addition, previous studies also regarded patients with recurrent MPE as those experiencing at least one additional pleural procedure after the first thoracentesis and their pleural effusion was confirmed malignant.^31^, ^32^ Also, in the diagnosis of MPE, CT appears to add additional value, with improved characterization of pleural nodularity, diaphragmatic thickening, and evidence of metastatic disease in the lung, abdomen, and chest wall, which can further stratify risk and aid in diagnosis.^33^ Overall, in our study, after one treatment cycle with approximately 3−4 weeks, CT was used again to evaluate the treatment effect on pleural effusion for each patient. During the process, no additional pleural procedures were conducted. If there was an increase in pleural effusion compared to the baseline level, indicating further pleural procedures were required, it would be classified as recurrent pleural effusion. On the contrary, it would be diagnosed as non‐recurrent pleural effusion.

The main characteristics of this patient cohort were confirmed with pathology of primary lesion. Each patient's pleural effusion was obtained through thoracentesis. The cytological diagnosis of cancer cells in pleural effusion was confirmed by several pathologists. At the same time, the driving genes carried by cancer cells in pleural effusion were also tested, including *EGFR* mutations, Kirsten Rat Sarcoma Viral Oncogene Homolog (*KRAS*) mutations, *ALK* fusion, ROS Proto‐Oncogene 1, Receptor Tyrosine Kinase (*ROS1*) fusion, Rearranged During Transfection (*RET*) fusion, and Human Epidermal Growth Factor Receptor 2 (*HER2*) mutations. All participants provided handwritten confirmation notices. The study was approved by the Ethical Committee of Shanghai Public Hospital (K18‐089‐1)

### 2.2 Preparation of single‐cell suspension

After isolation, 15 mL of each pleural fluid sample were directly extracted for centrifugation and the cell precipitation was obtained. We used 1XPBS (HyClone) for cell resuspension. Subsequently, 40 µm porous filters (Corning) were used to filter the cells to remove impurities that may affect subsequent experiments. We used TC20 automated cell counter (Bio Rad) to count and determine the viability of cell suspensions.

### 2.3 Single‐cell RNA library construction and sequencing

Using PBS as the medium, we adjusted the concentration of cell suspension to 1 × 10^5^ cells/mL. We loaded the cell suspension onto the Microfluidics chip (GEXSCOPE Single Cell RNA seq Kit, Singleron Biotechnology), and carried out according to the operating instructions of the manufacturer. The method of library construction and sequencing was consistent with the way that our previously published article performed.^34^

### 2.4 Expression matrix acquisition and quality control

We used the scopetools method (https://anaconda.org/singleronbio/scopetools) to analyse the original data and the expression matrix was obtained. The specific analysis steps and parameters were consistent with the methods we previously published in our article.^34^ At the same time, we conducted quality control on the data. We removed cells with gene expression level less than 200 or greater than 5000. We also removed cells with 30 000 UMIs and mitochondrial components greater than 30%.

### 2.5 Data integration, cell clustering, and cell types annotation

We used the Seurat 4.3.0 process to perform dimensionality reduction and integration analysis on the data. Through the FindVariableFeatures and SelectIntegrationFeatures functions, we identified the features located in each data file for subsequent data integration. Then, we used the FindIntegrationAnchors function to identify anchors in the data file for data integration. Finally, we used the IntegrateData function to integrate 16 data files. We standardized each data matrix file by using the NormalizeData and ScaleData functions. We conducted dimensionality reduction on the integrated data and identified cell subpopulations using the FindClusters function. The Uniform Manifold Approximation and Projection (UMAP) and t‐Distributed Stochastic Neighbour Embedding (t‐SNE) methods were used as visualization methods for cell clustering. Through the identification of cell subpopulations, we obtained a total of 14 cell subpopulations. Through SingleR_ 2.2.0 combined with the manual annotation method of PanglaoDB database, we annotated 7 different cell types (cancer cells, macrophages, monocytes, fibroblasts, T cells, dendritic cells (DCs) and Pre‐B cells). We identified conservative gene markers for each cell subpopulation using the FindAllMarkers function. Each cell subgroup exhibited its own significant genetic markers. The differential genes between observation groups or between subgroups were identified using the FindAllMarkers function.

### 2.6 Transcription factor regulatory network analysis

We used Chip‐X enrichment analysis to predict transcription factors that regulate genes. This analysis method was detailed in the literature.^35^ In short, we used 27 genes related to metastasis and *CLDN4* as input variables for prediction. This prediction algorithm was based on past CHIP seq data and listed the most reliable regulatory transcription factors based on scoring, namely E74 Like ETS Transcription Factor 3 (*ELF3)*, KLF Transcription Factor 5 (*KLF5)*, and Ovo Like Transcriptional Repressor 1 (*OVOL1)*. And a transcription factor network related to regulating these genes was obtained.

### 2.7 Differentially expressed gene analysis

We analysed the data of *ELF3*‐knockout (KO) (GSE148105) to determine the regulatory relationship between *CLDN4* and *ELF3*. In short, we used limma_ 3.56.2 to conduct differential gene analysis between the KO group and the wild‐type group. We obtained genes with significant upregulation and downregulation, and the evaluation criteria constricted that the absolute value of Log2Fc was greater than 1.5 and p.adj was less than 0.05.

### 2.8 Clustered regularly interspaced short palindromic repeats (CRISPR)‐CRISPR‐associated protein 9 (Cas9)‐guided KO (CRISPR‐KO) data analysis

To further determine the phenotype of *CLDN4*/Claudin‐7 (*CLDN7*) genes in tumour cells, vascular endothelial cells, and stem cells, we used CRISPR‐KO data to further screen the effects of these two genes on cell growth and proliferation in cell lines. We further validated the effects of knocking out *CLDN4* and *CLDN7* genes on cell growth and proliferation in A549, NCIH1975, 143B, HUES62, HCT‐15, and BxPC‐3 cell lines using the BioGRID ORCS database.^36^ Due to different gene KO platforms, the results were displayed as significant or insignificant KO.

### 2.9 Correlation analysis of single‐cell gene expression

In order to determine the genes positively correlated with *CLDN4* gene expression, we conducted a correlation analysis of gene expression in cells in the C1 cell subgroups, using *CLDN4* as the comparative object. In short, we obtained the expression matrix of C1 cell subpopulations and calculated the Pearson correlation coefficient. At the same time, we calculated the *p*‐value of the calculated correlation coefficient, and took a *p*‐value less than 0.05 as significant.

### 2.10 Gene functional annotation and signal pathway enrichment

In order to further analyse the differential genes between different observation categories (non‐recurrent and recurrent), we used the gene set enrichment analysis (GSEA) method to annotate the signalling pathways of genes that were upregulated or downregulated in expression.^37^ Meanwhile, we used clusterProfiler_ 4.8.1 to perform gene ontology (GO) annotation on differential genes.

### 2.11 Pseudo time series and cell trajectory analysis

We used the Monocle2 method to characterize cell trajectories and conducted quasi temporal analysis. In short, in this study, we analysed the differentiation status of C2 and C3 monocyte subpopulations, further identified the differentiation characteristic genes of these two cell subpopulations, and annotated these genes. The specific analysis steps and parameters were consistent with the methods we previously published in our article.^34^

### 2.12 Cell–cell interaction analysis

We used the Cellchat method to conduct a detailed analysis of cellular communication associations on the target cell subpopulations we interested.^38^ The target cell subpopulations of interest included C1 tumour cell subpopulations, C2, C3 monocyte subpopulations, and C6 fibroblast subpopulations. We first classified by observation categories (non‐recurrent and recurrent). Moreover, we associated and enriched all cellular and relevant cellular communication pathways according to the direction of cellular communication (sending or receiving). Among them, the *VEGF*‐Kinase Insert Domain Receptor (*KDR*) and Macrophage Migration Inhibitory Factor (*MIF*)‐CD74/CD44 signalling pathways had the greatest impact on the function of our target cell subsets. We also presented and elaborated on the senders, receivers, mediators, and influencers of signals according to the analysis process.

### 2.13 Real‐time polymerase chain reaction (PCR) analyses

Total RNAs of 64 MPE samples were prepared and retro‐transcribed into first‐strand cDNA using the first‐strand synthesis system following the manufacturer's protocol (B639251, Sangon biotech, Shanghai). The primers of four genes we identified, including *CLDN4*, *ELF3*, *TACSTD2*, and *EPCAM*, were designed and synthesized (Sangon biotech, Shanghai). Real‐time PCR was performed and the experiments were done in quadruplicates. Primers used for PCR were presented in the primers table. Data were calculated as mean ± SEM (Table 1).

**TABLE 1:** The designs of primers used for real‐time PCR.

Primers table	
*ELF3*	
Forward primer	GGCCGATGACTTGGTACTGAC
Reverse primer	GCTTGCGTCGTACTTGTTCTTC
*CLDN4*	
Forward primer	TGGGGCTACAGGTAATGGG
Reverse primer	GGTCTGCGAGGTGACAATGTT
*EPCAM*	
Forward primer	AATCGTCAATGCCAGTGTACTT
Reverse primer	TCTCATCGCAGTCAGGATCATAA
*TACSTD2*	
Forward primer	ACAACGATGGCCTCTACGAC
Reverse primer	GTCCAGGTCTGAGTGGTTGAA
18S RNA	
Forward primer	ACCCGTTGAACCCCATTCGTGA
Reverse primer	GCCTCACTAAACCATCCAATCGG

### 2.14 Statistical analysis

The Mann–Whitney U test was used for inter‐group comparisons to compare differences. Survival analyses based on Kaplan–Meier method were performed and the further Cox proportional‐hazards model was built. All statistical significance criteria were *p‐*values less than 0.05.

## 3 RESULTS

### 3.1 Cancer cells, monocytes, and fibroblasts displayed prominent variations between non‐recurrent and recurrent MPE

**FIGURE 1:**
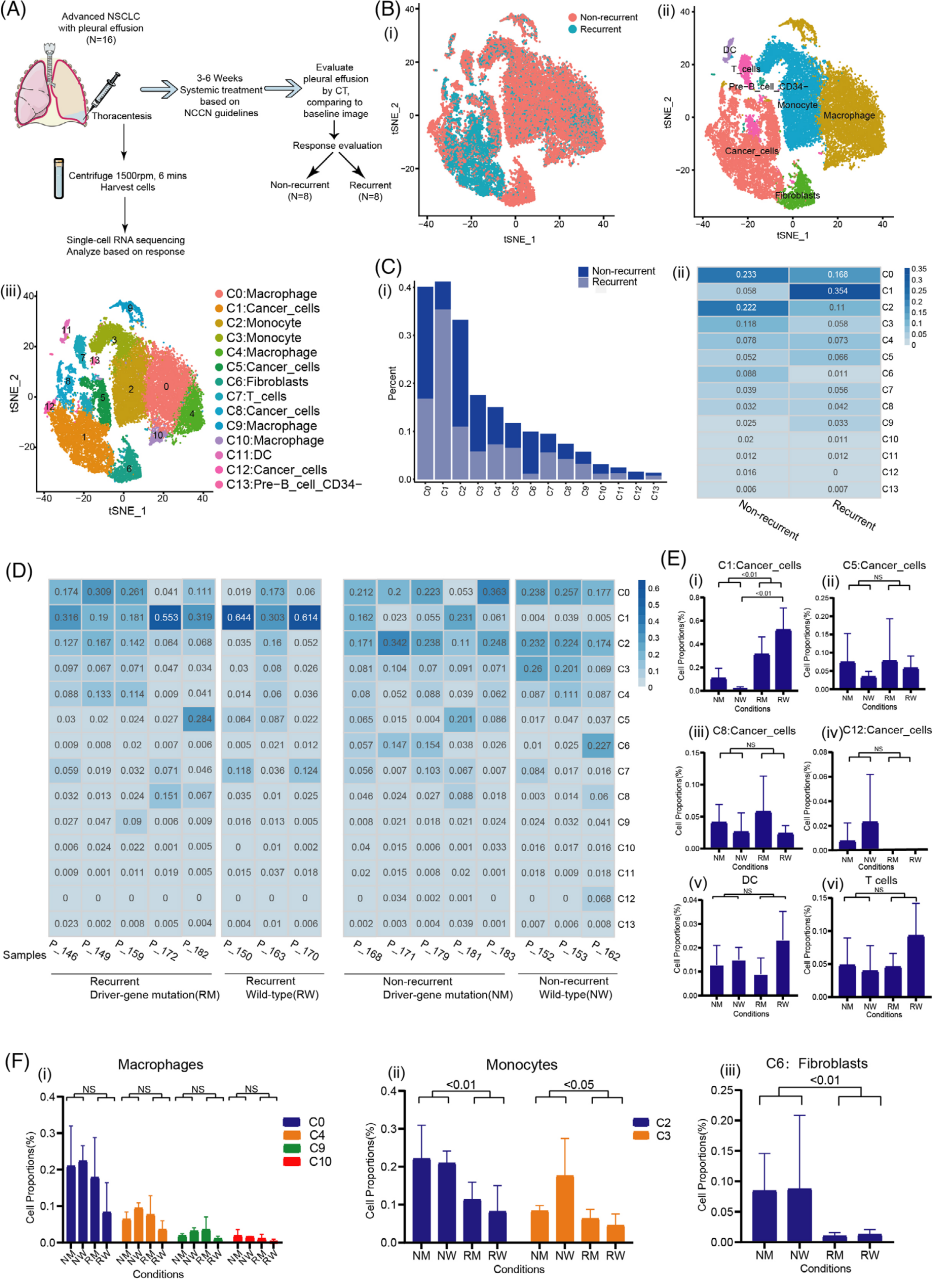
The C1 cluster cancer cells with highest percentage were captured in the recurrent MPE. (A) Schematic figure of sample collection and data acquisition. (B‐i) t‐SNE dimensional reduction of all the cells from 16 samples. (ii) Cell type annotation in the cells. (iii) Discrete cell type annotation in the C0‐C13 clusters. (C‐i) The cell percentage in non‐recurrent and recurrent cell clusters. (ii) Total cell percentage in the non‐recurrent and recurrent cell clusters. (D) Discrete cell percentage across four groups of samples and cell clusters. (E) Inter‐group comparison of cell percentage in cancer cells, DCs and T cells. (F) Cell percentage inter‐cluster comparisons in (1) macrophages, (2) monocytes and (3) fibroblasts. t‐SNE: t‐Distributed Stochastic Neighbor Embedding; DCs, dendritic cells; MPE, malignant pleual effusion.

A cohort of 16 patients with pleural effusion and subsequent treatments were included in the study. To discover the cell atlas which manifested detailed information of MPE, thoracentesis was performed and pleural effusions were harvested. The baseline MPE samples were collected, followed by matching therapeutics for 3−6 weeks. CT scan was performed to determine the status of recurrence. The samples were classified into non‐recurrent (*n* = 8) and recurrent (*n* = 8) groups according to the treatment response compared with baseline level. The scRNA‐seq was performed on the baseline samples (Figure 1A). Cells displayed different distribution between non‐recurrent and recurrent groups visualized by t‐SNE (Figure 1B‐i). Subsequently, cells were annotated by the conserved cell markers and clustered into 7 main populations, including cancer cells, macrophages, monocytes, fibroblasts, DCs, T cells and pre‐B cells (Figure 1B‐ii). The main cell groups were further classified into 14 individual clusters according to internal cellular characteristics inferred by t‐SNE. The cell clusters C1, C5, C8, and C12 were cancer cell clusters, which distributed much more in recurrent MPE generally compared with the non‐recurrent group (Figure 1B‐iii). Among all 14 clusters, C1 was noticed as an evident proportion of recurrent cells, and possessed the largest amounts of cells compared with other clusters (Figure 1C‐i,ii). C5, C8, C12 held different proportion of recurrent cells and possessed differently in cell number. All four cancer cell clusters jointly held the majority of the cell abundance among all clusters and therefore might exhibit dominant functions in recurrent MPE (Figure 1C‐i,ii).

To further verify whether the distribution of 14 clusters was influenced by potential confounders, we formatted cell proportions on a cell cluster‐samples basis. Samples were grouped by gene mutation status and the recurrent status of MPE. Four groups were attained, namely, recurrent driver‐gene mutation (RM) group, recurrent wild‐type (RW) group, non‐recurrent driver‐gene mutation (NM) group and non‐recurrent wild‐type (NW) group (Figure 1D). As expected, C1 presented a universal distribution in all samples while C12 diminished in most recurrent samples, and comparative analysis showed no significant differences between C5 and C8 among these groups (Figure 1D,E‐i‐v). We noticed that C1 showed a significantly larger cell proportion in RM group compared with NM group, which was also seen between RW and NW groups (Figure 1E‐i). Therefore, we concluded that the statistical variation in proportion of C1 cells was independent of gene mutation status and could be a universal marker for recurrent MPE in advanced NSCLC. In addition, significant differences were neither to be found in DCs, T cell clusters (Figure 1E‐v,vi) nor macrophage clusters (Figure 1F‐i). However, the proportion of monocyte clusters (C2, C3) and fibroblast cluster (C6) decreased markedly in recurrent MPE compared with the non‐recurrent group (Figure 1F‐ii,iii). In brief, cancer cluster C1 might play a role in the recurrent status of MPE since C1 was significantly enriched in recurrent MPE compared with the non‐recurrent group, while possessing the largest cell number and proportion among all clusters in recurrent MPE.

### 3.2 Comparative gene expression analysis deciphered C1 cluster signatures among heterogenous cell population

**FIGURE 2:**
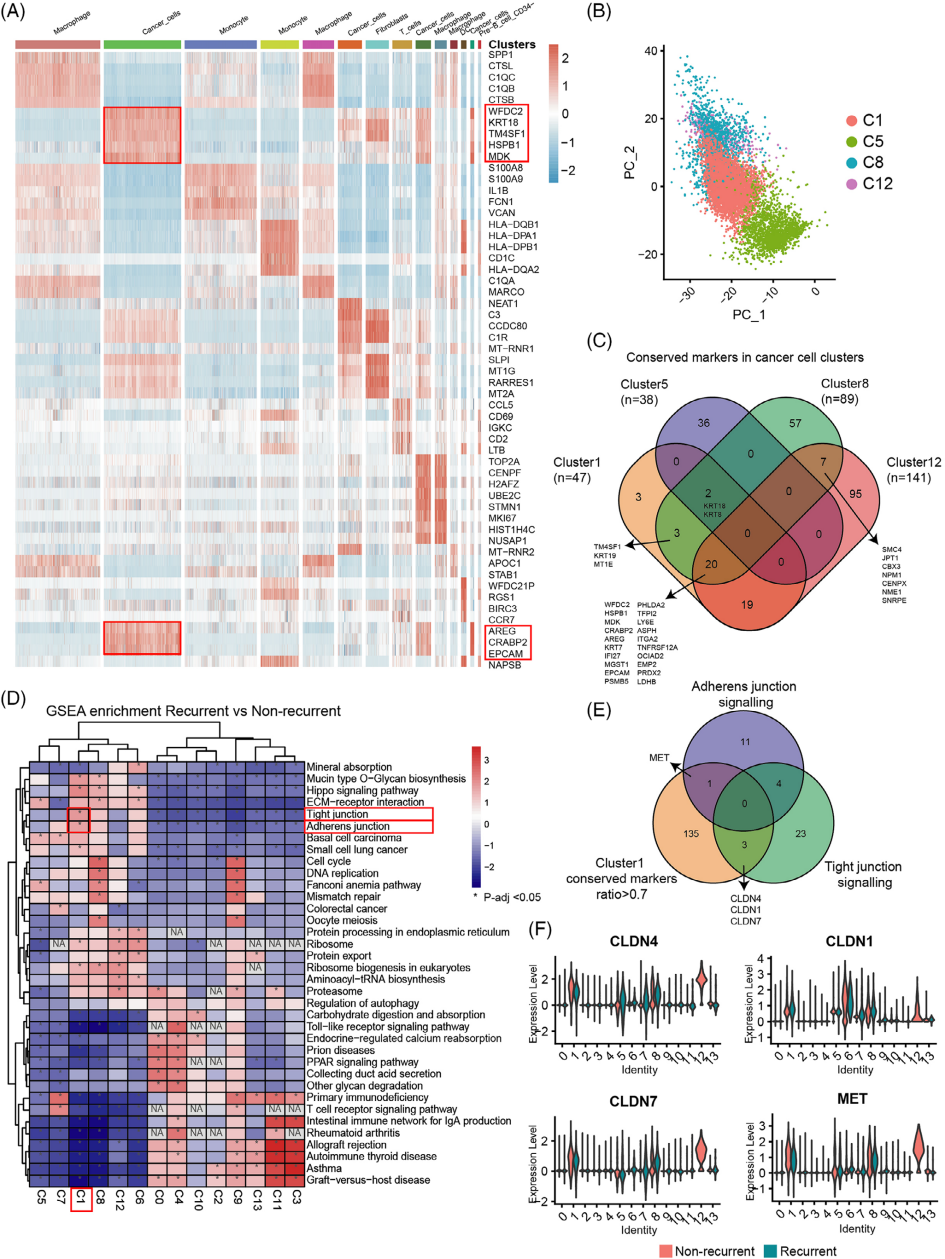
Molecular identification and functional annotation of gene signatures of cancer cells in MPE. (A) Heatmap illustrating conserved markers expressed across 14 cell types. (B) PCA dimensional reduction of cancer cells C1, C5, C8 and C12. (C) The overlapping gene signatures in C1, C5, C8 and C12 clusters. (D) GSEA enrichment of differentially expressed genes between recurrent versus non‐recurrent in cell clusters. (E) The overlapping genes in 3 genesets. (F) The expression of CLDN4, CLDN1, CLDN7 and MET across all 14 clusters. PCA, principal component analysis; GSEA, gene set enrichment analysis; MPE, malignant pleural effusion.

To further explore the conserved genes expressed in each individual population, we identified the genes closely related to each cluster (Figure 2A). Among them, WAP four‐disulfide core protein2 (*WFDC2*), Keratin 18 (*KRT18*), *EpCAM* were the top three genes with the highest upregulation of expression in cluster C1. These genes were associated with tumour migration and invasion.^39^, ^40^, ^41^ These traits illustrated that C1 cluster tended to have higher metastatic phenotype and might contribute to the recurrence of MPE. We used the principal component analysis (PCA) to further distinguish the four cancer cell clusters. Except for C12, the other three clusters were basically separated and the overlaps showed incompletely convergent and indicated their traits in common (Figure 2B). Then, the conserved genesets were compared and merged to find the similarities and differences (Figure 2C). The overlaps manifested the common marker genes. *KRT18* and Keratin 8 (*KRT8)* were presented in C1, C5, and C8. *WFDC2* and *EpCAM* along with several genes were clustered in C1, C8 and C12. There were also overlaps among C1, C8, and C12. C1 possessed the largest proportion of recurrent cells, and it was suggested that more focuses should be given to the C1‐related genes while exploring for diagnostic markers.

Subsequently, we performed the GSEA on 14 individual clusters between non‐recurrent and recurrent categories (Figure 2D). Significant enrichment results of signalling pathways on tight junction and adherens junction were found in C1, which implied the unique characteristic of C1 cluster and was consistent with the function of previously found genes, such as *KRT18*, *KRT8*, *WFDC2* and *EpCAM*. Then, we aimed to investigate the genes resided in the overlap of C1 conserved markers, adherens junction signalling and tight junction signalling. *CLDN4*, Claudin‐1 (*CLDN1)*, *CLDN7* genes were subsequently observed in the overlap of C1 and tight junction, and Mesenchymal to Epithelial Transition Factor (*MET)* gene was found in the overlap of C1 and adherens junction (Figure 2E). All four genes could be reliably detected in C1 (Figure 2F). The expression of these genes could also be detected in several clusters, but mainly in cancer cell clusters. Thus, the recurrent MPE might be defined by the potential diagnostic gene panel consisting of these four genes.

### 3.3 *ELF3*‐*CLDN4* feedback loop mediated an ameboidal‐type cell migration related metastatic tumour phenotype

**FIGURE 3:**
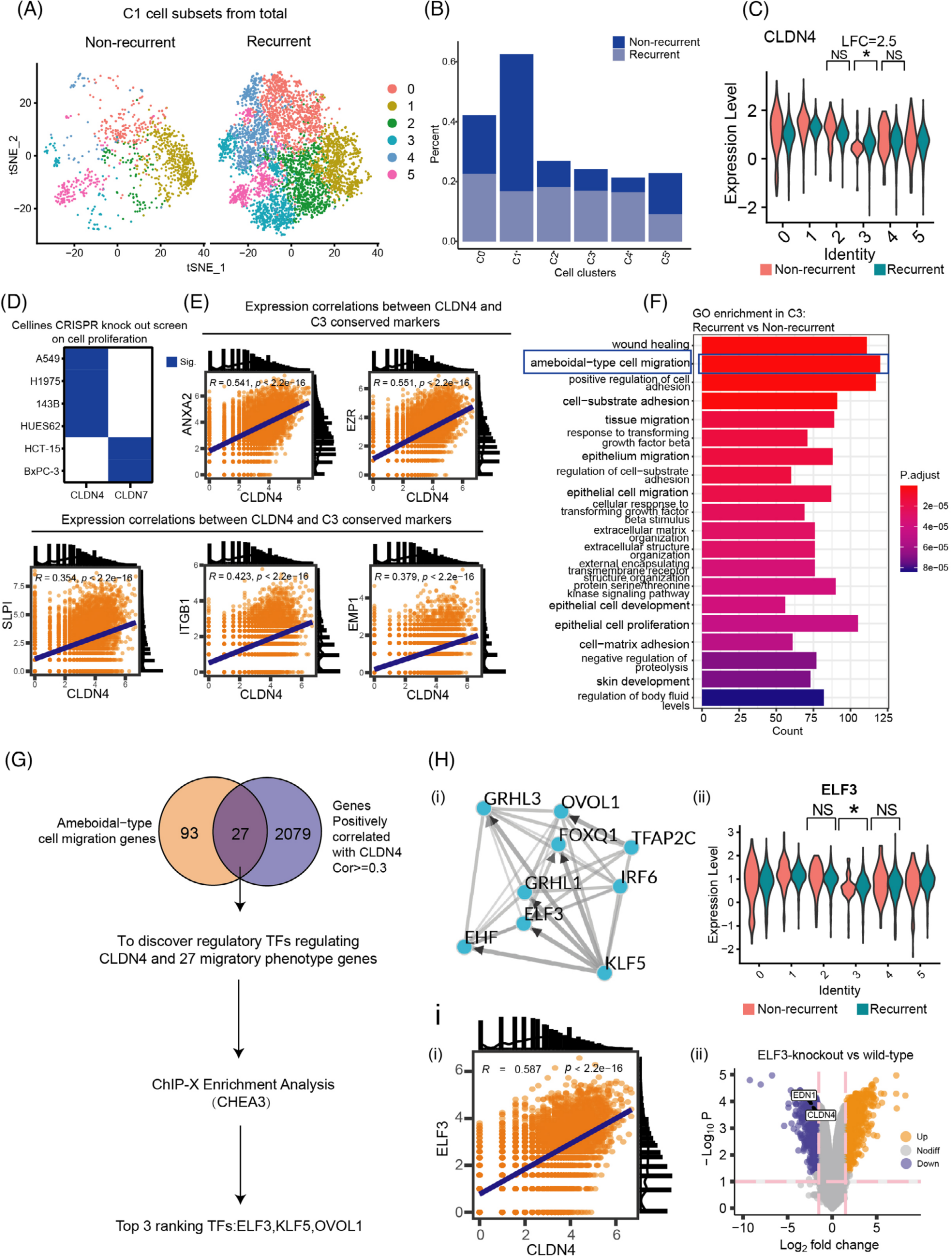
CLDN4, identified as a biomarker for recurrent MPE, was correlated with cancer cell proliferation and was regulated by transcription factor ELF3. (A) t‐SNE plot illustrated re‐clustered clusters C0‐C5 of C1 cluster from the 14‐cell clusters. (B) Cell percentage of non‐recurrent and recurrent in the re‐clustered C1 cluster. (C) CLDN4 expression across 6 clusters. (D) Clustered regularly interspaced short palindromic repeats‐cas9‐guided knock out (CRISPR‐KO) data analysis indicated cell proliferation status of CRISPR‐knockout screening in 6 different cell lines. (E) The correlated gene with CLDN4 in the C3 cluster. (F) GO enrichment of differentially expressed genes between recurrent versus non‐recurrent in the C3 cluster. (G) Working model illustrating the procedure of screening regulatory transcriptional factors based on transcription factor regulatory network analysis. (H‐i) Regulatory transcriptional factors network based on transcription factor regulatory network analysis. (ii) ELF3 expression across 6 clusters. (I‐i) Correlation analysis of gene expression between CLDN4 and ELF3 in C3 cluster, described above. (ii) The differentially expressed genes between ELF3‐knockout and wildtype group inferred by differentially expressed gene analysis. t‐SNE, t‐Distributed Stochastic Neighbor Embedding; GO, gene ontology; MPE, malignant pleural effusion.

C1 was a heterogenous subset of cancer cells expressing diverse conserved markers, requiring for a further precise classification. To define the sub‐level gene‐cell signatures, C1 was further clustered into six subgroups. Consistent with the above results, the number of these cells in recurrent MPE was significantly higher than that in non‐recurrent MPE (Figure 3A). The proportion of each sub‐cluster derived from C1 was explicitly presented, among which C2, C3, C4 showed a noticeably dominant proportion in recurrent MPE group (Figure 3B).

Then the expression levels of *CLDN1*, *CLDN4*, *CLDN7* and *MET* were evaluated in the six clusters of C1 cell subset. Our results demonstrated that the expression level of *CLDN4* and *CLDN7* (data not shown) was elevated in C3 but not in C2 or C4 subset (Figure 3C). Through the /CRISPR‐Cas9 KO cell proliferation screening in six cell lines, *CDLN4* was found to be significantly related to cell proliferation in lung cancer cells (A549, NCIH1975) and others (143B, HUES62). *CLDN7* was screened out due to no specific cell proliferation phenotype was observed in lung cancer cell lines (Figure 3D). Moreover, the expression correlation between *CLDN4* and the conserved markers of C3 were validated. Genes like Annexin A2 (*ANXA2*), Ezrin (*EZR*), Secretory Leukocyte Peptidase Inhibitor (*SLPI*), Integrin Subunit Beta 1 (*ITGB1*) and Epithelial Membrane Protein 1 (*EMP1*), which were extracellular matrix related genes, showed a positive correlation with *CLDN4* (Figure 3E). Previous studies have suggested that the extracellular matrix related genes determined the oncogenesis in lung cancer, and could be used as predictive indicators.^42^, ^43^ The GO enrichment analysis was performed to identify the transcriptomic signature between recurrent verses non‐recurrent status in C3 cluster, indicating the typical characteristics of the expression of ameboidal‐type cell migration related genes among C3 subset in recurrent MPE (Figure 3F). Ameboidal‐type cell migration was reported to be one of the typical migration modes of the invasive tumour cells. This mode augmented the aggressive and metastatic behaviour of tumour cells and promoted their survival, invasion and colonization.^44^ Meanwhile, to discover the expression pattern of *CLDN4* and its regulatory factors, we conducted ChIP‐X enrichment analysis 3 (ChEA3) analysis. The ameboidal‐type cell migration genes and the *CLDN4*‐positively correlated genes were overlapped, and subsequently 27 genes were acquired for downstream analysis. Through this algorithm, transcription factors (TFs), that regulated the *CLDN4* expression and ameboidal‐type cell migration related genes, were able to be identified from these genes. TFs were predicted and ranked, and the top three ranking TFs were *ELF3*, *KLF5*, and *OVOL1* (Figure 3G). The interconnected network of TFs was constructed (Figure 3H‐i). Among them, *ELF3* was found to be expressed among all cell subsets and was significantly enriched in C3 subset among current MPE compared with non‐recurrent group, which marked the unique characteristics of C3 and associated *CLDN4* with *ELF3* (Figure 3H‐ii). The expression of *ELF3* and *CLDN4* was positively correlated, indicating its regulatory role (Figure 3I‐i). And when *ELF3* was knocked out, the expression of *CLDN4* decreased simultaneously (Figure 3I‐ii). These findings emphasized the regulatory role of *ELF3* on *CLDN4*, as *CLDN4* was inclined to be a novel candidate for clinical prediction or diagnosis of recurrent MPE.

### 3.4 Recurrent MPE‐related *CLDN4* expression postulated consistent signatures that could be involved in hypoxia‐induced angiogenesis independent of gene mutation status

**FIGURE 4:**
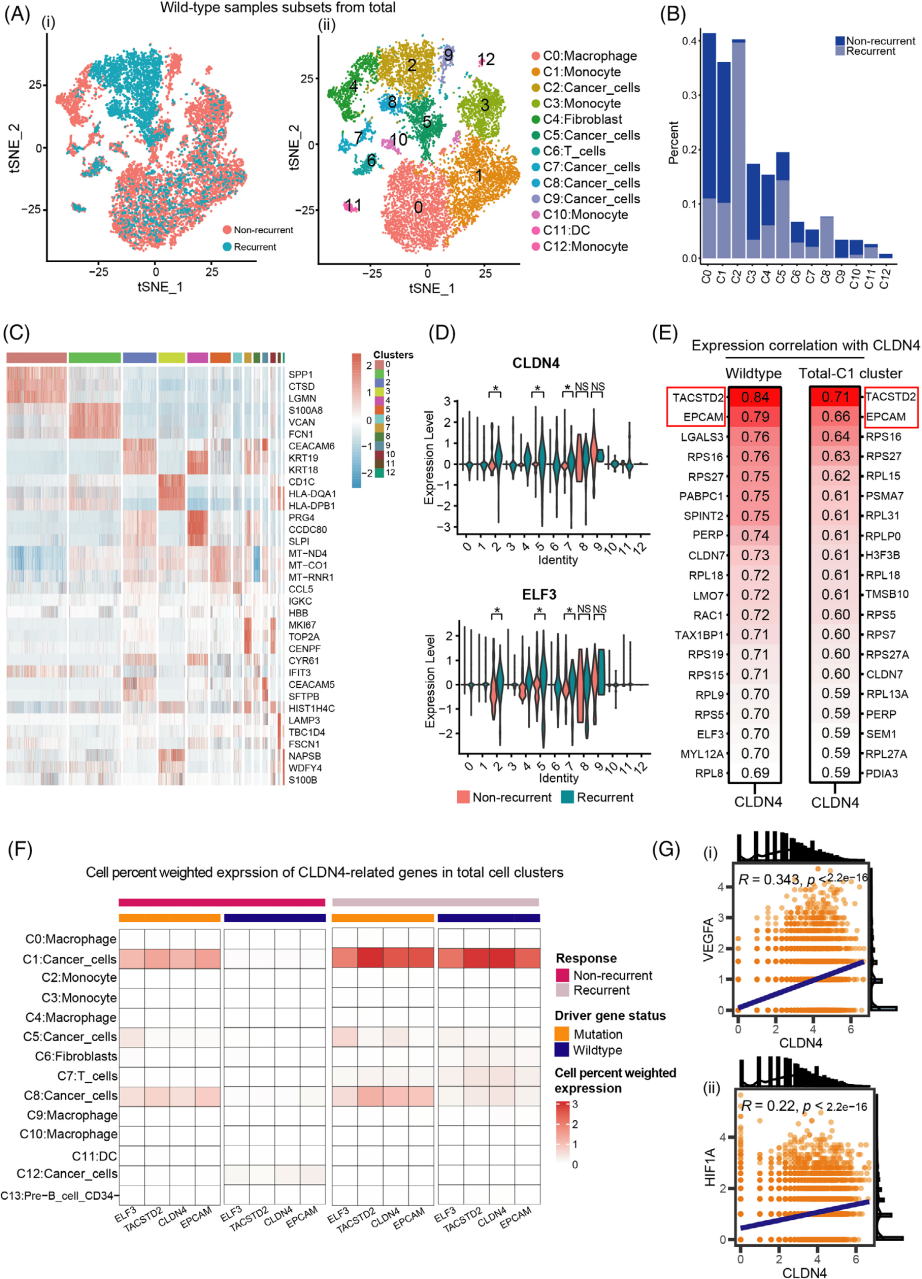
CLDN4, as a biomarker for recurrent MPE, was independent of driver gene mutation status. (A) t‐SNE plot of re‐clustered wildtype samples from the total cell clusters, grouped by (i) sample types and (ii) cell type annotation. (B) Cell percentage of the non‐recurrent and recurrent groups in the re‐clustered C1 cluster. (C) Heatmap illustrating conserved markers expressed across re‐clustered 13 cell clusters. (D) CLDN4 and ELF3 expression in the 13 cell clusters. (E) Genes correlated with CLDN4 in the subset of wildtype cells and C1 cluster. (F) The cell‐percentage weighted expression of CLDN4‐related genes in total cell clusters. (G‐i,ii) VEGFA and HIF1A were expressed positively correlated with CLDN4. t‐SNE, t‐Distributed Stochastic Neighbor Embbedding; MPE, malignant pleural effusion.

As mentioned above, we have illustrated *CLDN4* as a potential diagnostic marker expressed on cancer cells for recurrent MPE. However, whether the driver‐gene mutation status affected the expression of *CLDN4* remained uncertain. Thus, the samples with no driver‐gene mutations were investigated. Cells were re‐clustered and 13 clusters were attained eventually, with a distinct distribution of clusters in recurrent group (Figure 4A‐i). Among them, C2, C5, C7, C8, and C9 were clustered as cancer cell subsets (Figure 4A‐ii). In total 13 clusters, C2, C5, and C8 manifested dominant proportion of recurrent cells compared to others (Figure 4B). Conserved markers closely related to each cluster were identified, which was consistent with the whole sample clusters. C2 expressed *KRT18* similar to total C1, and C4 expressed Coiled‐Coil Domain Containing 80 (*CCDC80*) identical with total C5 (Figure 4C). These conclusions supported the transcriptomic similarity between the wild type and the total MPE samples. Then, we verified the expression pattern of *CLDN4* in the wild‐type samples. *CLDN4* and *ELF3* expression was testified in all clusters and statistically significant increased expression of them were observed in cancer cell clusters C2, C5, and C7 (Figure 4D), indicating the similar expression status discovered in total cells. To identify the genes that correlated with *CLDN4* in wild‐type and total cluster C1, we found that the top‐two related genes, *TACSTD2* and *EpCAM*, were accordant (Figure 4E). *TACSTD2* and *EpCAM* were previously reported to be associated with cell adhesion and metastasis of tumor,^45^ implying the effect on recurrent MPE accompanied with *CLDN4*. These results indicated that *CLDN4*, *TACSTD2*, *EpCAM*, and *ELF3* were closely related gene signatures that could present as potential diagnostic markers for recurrent MPE. In the cell percent weighted expression analysis, total clusters were classified into non‐recurrent and recurrent groups and were further classified into the mutation and wild‐type groups. Although mild expressions were demonstrated in total cancer cell cluster C1 and C8 in non‐recurrent group, the *CLDN4* expression along with *ELF3*, *TACSTD2* and *EpCAM* could only be seen in recurrent cells of total C1. The expression patterns of the selected *CLDN4*‐related four genes in total C1 cluster was independent of gene mutation status (Figure 4F). Further expression correlation analysis identified positive relations of *CLDN4* with Vascular Endothelial Growth Factor A (*VEGFA)* and Hypoxia Inducible Factor 1 Subunit Alpha (*HIF1A)* (Figure 4G‐i,ii), which were both factors that associated with angiogenesis and hypoxia,^46^, ^47^ and could be the mechanistic understanding of facilitating the recurrence of MPE.

### 3.5 Hypoxia‐induced angiogenesis and *MIF* signalling were the potential mechanistic explanations of MPE recurrence

**FIGURE 5:**
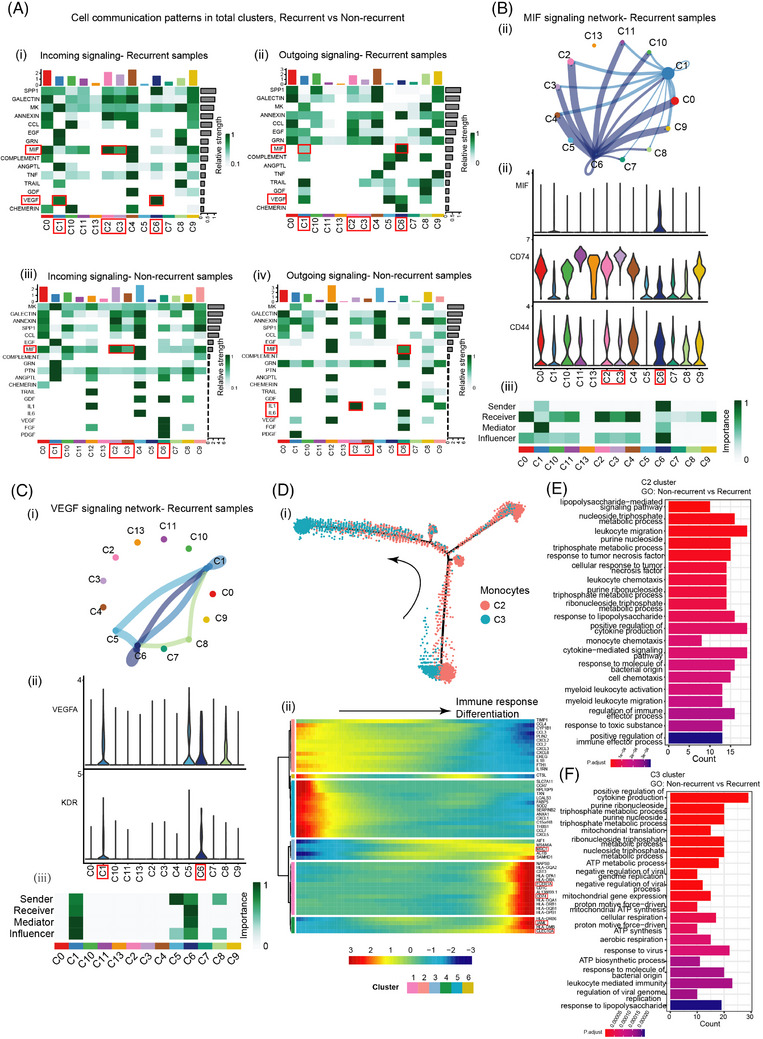
Cellular interaction analysis indicating MIF signalling and VEGFA signalling were key influencer in the recurrent MPE. (A) Cell communication patterns in recurrent samples (i,ii) and non‐recurrent samples (iii,iv). (B) MIF signalling in the recurrent samples. (i) Interaction pattern of MIF signal among all cell clusters. (ii) MIF, CD44, and CD74 expression among all the cell clusters. (iii) Summary of signal directions and significance. (C) VEGF signalling in the recurrent samples. (i) Interaction pattern of VEGFA signal among all cell clusters. (ii) VEGFA and KDR expression among all the cell clusters. (iii) Summary of signal directions and significance. (D) Deciphering the differentiating signatures by trajectory inferences. (i) Cell trajectory of C1 and C3 monocyte. (ii) Gene signatures in the process of monocytes differentiation. (E) GO enrichment of differentially expressed genes between recurrent versus non‐recurrent in the C2 cluster. (F) GO enrichment of differentially expressed genes between recurrent versus non‐recurrent in the C3 cluster. GO, gene ontology; MPE, malignant pleural effusion.

A cell communication network analysis of incoming and outgoing signallings were performed, based on the samples from non‐recurrent and recurrent group (Figure 5A‐i‐iv). In recurrent samples, the monocyte clusters C2 and C3 received signals of *MIF* signalling mainly sent by fibroblast cluster C6, and they also received signals of *MIF* pathway sent by cancer cell cluster C1 (Figure 5A‐i,ii). Meanwhile, C1 directly sent signals to C6 via *VEGF* pathway (Figure 5A‐i,ii). While in non‐recurrent samples, C1 no longer participated in *VEGF* and *MIF* signal transduction with C2, C3, and C6, while C6 sent signals to C2 and C3 via *MIF* pathway, and C2 sent out signals through Interleukin 1 (*IL1*) pathway, which could be related to anti‐tumoral immunity and pleural effusion control (Figure 5A‐iii,iv). As previous data showed that C2 and C3 monocytes declined in the recurrent group compared with non‐recurrent group, C1 participated in signalling communications with C2, C3 and C6 in recurrent samples compared with non‐recurrent samples. *MIF* signalling network displayed profound associations between C1 and other clusters in recurrent samples (Figure 5B‐i). From previous studies, *MIF* would combine with *CD44* and *CD74* molecules and apply their function as monocyte inhibitory signaling.^48^ We discovered C2 and C3 had a high expression level of *CD44* and *CD74* compared with C6 (Figure 5B‐ii). Among these clusters, C6 represented as signal sender and influencer aiming at C2 and C3, which were also receivers. C1 participated in the signalling by mediated the signalling. As a result, the number of monocytes in recurrent category was suppressed and their inflammatory anti‐tumour effects were impaired (Figure 5B‐iii).

Simultaneously, C1 interconnected with C6 through *VEGF* signalling pathway (Figure 5C‐i). C1, C5, C6 concurrently participated in *VEGFA*‐*KDR* signalling. Among which, C1 and C6 were main senders and mediators. C5 showed mild sender effect as well (Figure 5C‐ii,iii). These results indicated that the cancer cell cluster C1 was involved in the regulation of *VEGFA* interaction among pleural effusion cells. That could in a way explained the reason why many clinical studies focusing on *VEGF*‐targeted therapy for NSCLC with MPE only received limited response.^49^, ^50^ Pseudo‐time analysis was performed on monocyte C2 and C3. The cell trajectory of C3 tended to differentiate into the terminal status (Figure 5D‐i), where they up‐regulated the expression of Mannose Receptor C‐Type 1 (*MRC1*) (Figure 5D‐ii). *MRC1* was an M2‐associated marker and took part in immune suppression,^51^ indicating that M2‐associated monocytes could induce recurrence of MPE. GO enrichment was used to precisely analyse the differentially expressed gene between non‐recurrent and recurrent MPE in C2 and C3. We found that the leukocyte migration related‐signalling and cytokine production related‐signalling were respectively highly enriched in the C2 and C3 cluster favouring non‐recurrent MPE (Figure 5E,F), suggesting that the inflammation might be switched on by the functional immune elements so that a non‐recurrent status could have reached. The above results suggested that monocyte cytokine production related signalling was closely associated with the non‐recurrent MPE, which essentially activated anti‐tumour immunity in pleural effusion. As the largest proportion of cells in pleural effusion, tumour cells were the core cause of recurrent pleural effusion. Therefore, identification of tumour cell markers in pleural effusion could provide a novel and supplementary diagnostic method for early prediction of recurrent pleural effusion.

### 3.6 Clinical correlation of *CLDN4* expression with MPE recurrence

**FIGURE 6:**
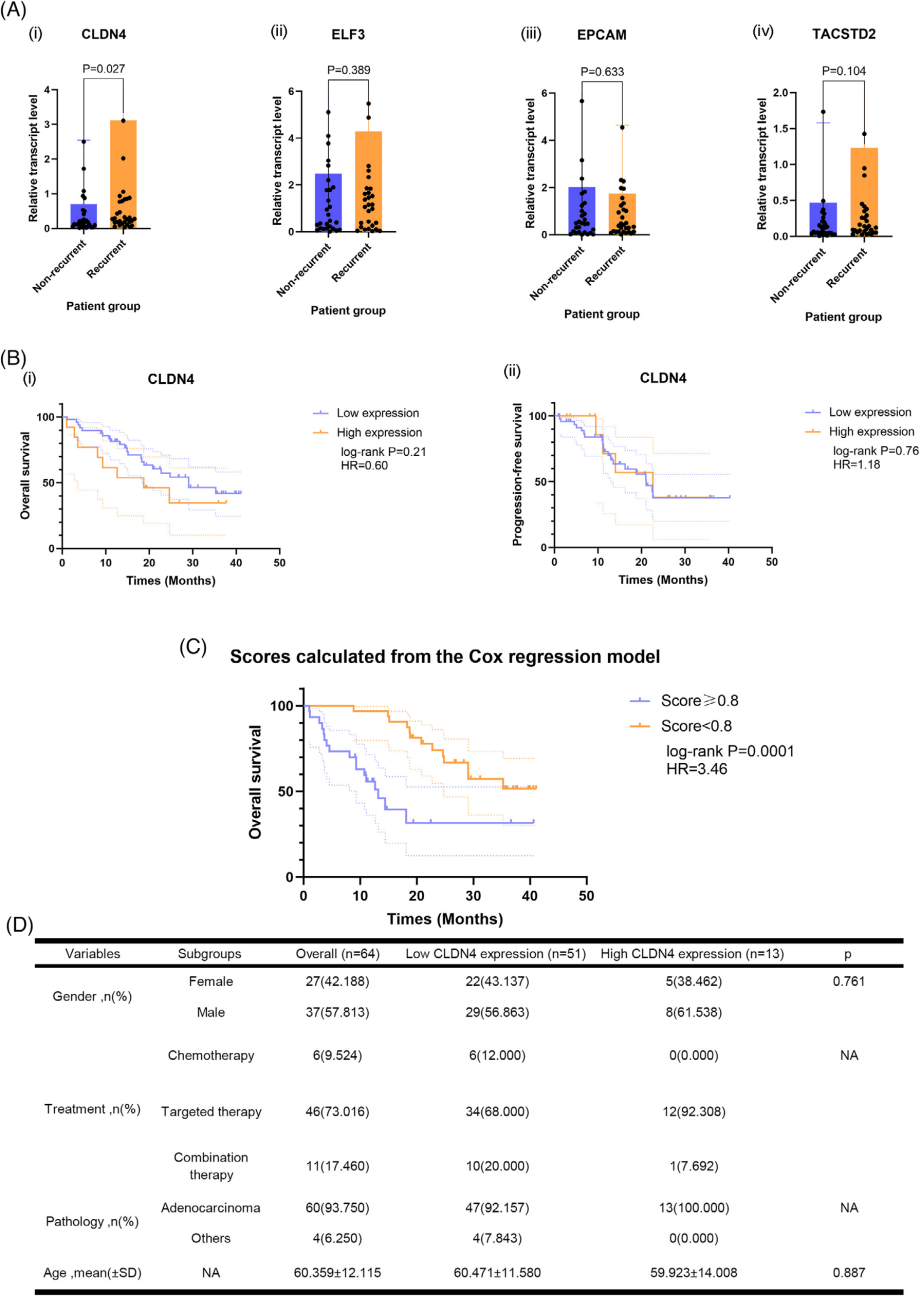
Clinical validation of CLDN4 as a diagnostic biomarker in predicting recurrent MPE. (A) The expression level of (i) CLDN4, (ii) ELF3, (iii) EPCAM and (iv) TACSTD2 in advanced NSCLC patients with non‐recurrent or current MPE in the validation cohort. (B‐i) The overall survival and (ii) progression‐free survival among advanced NSCLC patients with low or high expression of CLDN4. (C) The overall survival among advanced NSCLC patients with scores <0.8 or ≥0.8, which were derived from the Cox regression model consisting of CLDN4, ELF3, treatment strategies, and the pathology of disease. (D) The baseline characteristics of advanced NSCLC patients in the validation cohort. MPE, malignant pleural effusion; NSCLC, non‐small‐cell lung cancer.

To evaluate the association between the expression of the above four genes and the therapeutic response, we examined 64 patients diagnosed with advanced NSCLC with pleural effusion samples at baseline from Shanghai Pulmonary Hospital. The results of real‐time PCR indicated that the expression of *CLDN4* showed a significantly increased expression in the recurrent group versus the non‐recurrent group (*p *= 0.027) (Figure 6A‐i), while no significant difference was observed for *ELF3*, *EPCAM*, *TACSTD2* among the two groups (Figure 6A‐ii,iii,iv), further supporting the clinical value of *CLDN4* as a predictive marker for recurrent MPE. Meanwhile, we aimed to further explore the prognostic value of *CLDN4* through conducting additional survival analyses based on these patients. The results demonstrated that the high expression of *CLDN4* was associated with a trend towards worse overall survival (OS), though without marked difference (*p *= 0.21) (Figure 6B‐i). Nonetheless, we found that the expression of *CLDN4* could not discriminate the progression‐free survival (PFS) among the two groups (Figure 6B‐ii), which might be due to our small sample size along with potential bias. To further discriminate the survival difference regarding OS, the Cox regression model consisting of both gene expression information and clinicopathological characteristics was performed to identify the variables that could significantly influence the PFS, which might cooperate with *CLDN4* as a short‐term indicator for disease control and eventually influence the long‐term results of patients. Eventually, a model consisting of the *CLDN4*, *ELF3*, treatment strategies, and the pathology of disease, was successfully built and could be utilized to distinguish the OS significantly (*p *= 0.001) (cut‐off value = 0.8) (Figure 6C). The baseline characteristics of this cohort was also demonstrated (Figure 6D).

## 4 DISCUSSION

To the best of our knowledge, few studies proposed a clear and unified definition of recurrent MPE. Thus, the biological mechanism and representative markers underlying recurrent MPE still remains largely unknown. Nevertheless, in clinical practice, it is urgent to predict the recurrence of MPE prior to therapeutics, since recurrent MPE leads to impaired life quality and dampened prognosis. Many patients suffered from recurrent MPE,^29^ and the mechanisms between non‐recurrent and recurrent MPE could be different. Therefore, in this study, scRNA‐seq was conducted on both non‐recurrent and recurrent MPE samples. As an important tool for revealing gene expression patterns and single‐cell dynamic changes, single‐cell sequencing could decipher the transcriptome characteristics of cells with unprecedented resolution,^52^ which was quite appropriate for this study to analyse and reveal the heterogeneity of the microenvironment of non‐recurrent and recurrent MPE. In our study, *CLDN4* was verified as the potential predictive marker for recurrent MPE through a series of analyses, and its potential regulatory factor *ELF3* was identified as well. Further validation showed *CLDN4* expression was significantly higher in recurrent MPE samples than in non‐recurrent MPE samples in the extensive MPE cohorts from our centre. The prediction of recurrent MPE by the marker *CLDN4* might become a novel effective procedure, which would facilitate the management strategy for subsequent MPE at an earlier stage for those advanced NSCLC patients.

*CLDN4* is a tight junction protein which regulates paracellular permeability, cell polarity, and barrier function permanence.^53^ It is overexpressed and plays regulatory roles in multiple types of cancers.^54^, ^55^ In gastric cancer, *CLDN4* was found to enhance the proliferation, invasion and EMT of gastric cancer cells, and was reversed by miR‐596 and miR‐3620‐3p,^56^ while the overexpression of *CLDN4* induced EMT of ovarian cancer cells through *PI3K*/*Akt* and the EMT transcription factor Twist1 signal pathway similarly.^57^ Simultaneously, TGF‐β could induce glioblastoma mesenchymal transition through upregulation of *CLDN4* and nuclear translocation to activate *TNF‐α*/Nuclear Factor Kappa‐B (*NF‐κB)* signal pathway.^58^ Furthermore, previous studies also indicated that the expression of *CLDN4* could be utilized to distinguish the metastatic epithelial neoplasms in serous effusions and mesothelioma, including those from lung, breast, gastrointestinal tract, pancreas, ovary and primary serous papillary carcinoma of peritoneum.^59^, ^60^ Nonetheless, the biological function of CLDN4 on the development or progression of lung cancer still remained largely unknown, especially on its related comorbidity, MPE. In our study, besides discovering that *CLDN4* could be a predictive marker for recurrent MPE in advanced NSCLC, we further found that the expression level of *CLDN4* was positively correlated with the expression of *VEGFA* and *HIF1A*, and also validated that hypoxia‐induced angiogenesis and *MIF* signalling contributed mostly to the recurrence of MPE. The correlation between *CLDN4* and these signallings strongly suggested the role of *CLDN4* in the recurrence of MPE. Meanwhile, genes positively correlated with *CLDN4* in expression, such as *ANXA2*, *EZR* and *ELF3*, were also discovered in our study, which were also reported to play a role in tumour progression and might explain the role of *CLDN4* in a way. For instances, Ibrahim et al. suggested the *ANXA2* gene and its association with the cancer‐associated fibroblasts (CAFs) had significant effects on the MPE generation.^61^ Xu et al. discovered that *EZR* was also involved in ameboidal‐type cell migration like *ELF3*, and the tumour proliferation, invasion and migration were suppressed when *EZR* was knocked out.^62^ Kuang et al. pointed out that *ELF3* promoted tumour growth and metastasis by inhibiting microRNA‐485‐5p.^63^ Horie et al. also suggested that elevated *ELF3* expression level was linked to the tumour promotion traits in cancers.^64^ Overall, our study provided the initial data regarding the potential mechanisms of *CLDN4* contributing to the recurrence of MPE in advanced NSCLC, while further in‐depth experiments were required to perform to validate its specific mechanisms. Besides, due to the fact that our samples were collected at baseline, a test kit based on the expression of *CLDN4* might be developed in the future to assist physicians to identify NSCLC patients with high risk of recurrent MPE when they were treated initially. Furthermore, targeted drugs or antibodies on *CLDN4* might also had the potential to inhibit the development of recurrent MPE during the course of NSCLC.

On the other hand, different from tumour cells, the discrepancy between non‐recurrent and recurrent group in monocytes and fibroblasts revealed potential pathways which might affect the recurrence of MPE. *MIF* was originally found to inhibit macrophage motility.^65^ Subsequent study reported that *MIF* played a role in cell proliferation along with other tumour‐promoting processes.^66^ A phase I study investigated the efficacy of fully human recombinant antioxidized *MIF* antibody in advanced solid tumours, and reported an optimal response of stable disease (SD) in 26 patients. They suggested that *MIF* inhibitor could be used in the combination therapy for cancer.^67^ The complication of MPE was not mentioned in this study, but our study could provide a theoretical basis for the application of *MIF* inhibitors in MPE treatment, especially the recurrent one. Compared with the *MIF* pathway, *VEGF* pathway was more extensively explored in MPE studies. Du et al. reported that the efficacy of pleural bevacizumab combined with cisplatin was more significant than cisplatin alone.^50^ Nonetheless, Noro et al. suggested that in NSCLC patients with recurrent MPE, MPE still recurred among 20% of patients when they were administrated with anti‐vascular agents combined with chemotherapy, suggesting that the *VEGF* pathway had limitations in control of recurrent MPE,^68^ which could be explained by our findings that *MIF* signalling also played a significant role mediating the recurrence of MPE. Consequently, a rationale of the combinational use of both *MIF* and VEGF inhibitors in treating recurrent MPE in advanced NSCLC could be established, and further animal studies and clinical trials should be conducted to validate its efficacy and safety.

Limitations of this study were admitted, mainly including three aspects. First, the sample size used for scRNA‐seq and subsequent analysis was relatively small, thus the sampling bias was inevitable. In validation experiments based on the extensive cohort from our centre, we still used the total cells from malignant pleural effusion, which limited the strength of validation, since *CLDN4* was mainly expressed in a subset of metastatic lung cancer cells. Second, as for the cytology detection method we performed, real‐time PCR was used rather than flow cytometry (FCM). PCR could not value the expression on single‐cell level and lacked of differentiation of cell types compared to the FCM. However, PCR is currently the most cost‐effective and accessible method for clinical application, while FCM will increase the patient's medical expenses and demand for higher sample preservation requirements. Also, previous studies also indicated that the expression level of matrix metalloproteinase (MMPs) could increase in MPE,^69^ which might lead to the degradation of *CLDN4*. Thus, due to the complexity of MPE microenvironment, assessing the expression level of *CLDN4* might not be that ideal and available, while directly evaluating the expression level of *CLDN4* with real‐time PCR could be more stable and feasible clinically. Third, the cohort of patients included in the study was heterogeneous, and their subsequent treatments were different, which might directly determine the recurrence of MPE. For example, patients with adenocarcinoma and squamous cell carcinoma, and even further, adenocarcinoma patients with different oncogenic mutations, could have different genetic backgrounds, followed by different treatments. Nonetheless, as mentioned above, the small sample size restricted separate analysis under each clinical condition, and our findings could be considered as a commonality of cause of recurrent MPE shared by different conditions of lung cancer, which should be interpreted with cautious. Thus, further investigations with larger sample size investigating the recurrent MPE under specific clinical condition are warranted. Furthermore, the lack of attention of MPE caused by the diseases other than pleural mesothelioma restricted the molecular understanding of the MPE, leading to limited public resources related to the comorbidity of MPE with lung cancer. Thus, external validation based on public data was unavailable. Also, the regulatory role and biological functions of *CLDN4* in current study were mostly inferred by algorithms, and the subsequent validations should be conducted. Overall, our study posed the example for future studies exploring for the in‐depth understanding of recurrent MPE, and the regulatory role and mechanism of *CLDN4* should be thoroughly investigated through experiments.

## 5 CONCLUSION

Our study identified that *CLDN4* was a predictive marker of recurrent MPE among patients with advanced NSCLC.

## AUTHOR CONTRIBUTIONS

*Writing—original draft*: Xiaoshen Zhang, Xuanhe Wang and Yaokai Wen. *Formal analysis*: Xiaoshen Zhang, Yaokai Wen. *Investigation*: Xiaoshen Zhang,Yaokai Wen, Xuanhe Wang and Shen Chen. *Conceptualization*: Caicun Zhou and Fengying Wu. *Funding acquisition*: Caicun Zhou, Fengying Wu.

## CONFLICT OF INTEREST STATEMENT

The authors declared no conflicts of interest.

## FUNDING INFORMATION

Shanghai Key Clinical Specialty Construction Project—Respiratory Medicine (201912‐0552); National Key Clinical Program on Oncology.

## ETHICS STATEMENT

This work was carried out in accordance with The Code of Ethics of the World Medical Association (Declaration of Helsinki). This study was approved by ethical committee of Shanghai Pulmonary Hospital (No. L20‐351‐2). Written consents were obtained from all enrolled participants. This study was designed and reported complying with the REMARK Guidelines.^1^

## Data Availability

The single‐cell RNA sequencing data have been deposited to the China National Center for Bioinformation via the GSA‐Human repository with the dataset identifier HRA006761.
